# Identification and quantification of oral lymphatic vessel networks using optical coherence tomography (OCT)

**DOI:** 10.1364/BOE.595664

**Published:** 2026-08-25

**Authors:** Jiacheng Gu, Yilong Zhang, Simon Shepherd, Michaelina Macluskey, Zhihong Huang, Chunhui Li

**Affiliations:** 1 Healthcare Engineering, School of Physics and Engineering Technology, University of York, York YO10 5DD, UK; 2 School of Dentistry, University of Dundee, Dundee DD1 4HN, UK; 3 Centre for Medical Engineering and Technology (CMET), University of Dundee, Dundee DD1 4HN, UK

## Abstract

Lymphatic vessels play essential roles in immune regulation and tissue homeostasis, yet their distribution within the oral mucosa is incompletely quantified due to the lack of noninvasive, depth-resolved imaging tools. In this study, optical coherence tomography (OCT) combined with an optimized optical attenuation coefficient estimation (OAC) was used to segment and quantify lymphatic vessel networks in healthy and ulcerated human oral mucosa. The results showed descriptive depth-related patterns across the three oral regions (lip, hard palate, and buccal mucosa). The lip showed an overall increase across the measured depth intervals; the hard palate remained consistently low in mean lymphatic density with little variation, whereas the buccal mucosa increased in superficial intervals and gradually decreased at greater depths. In ulcerative lesions, lymphatic density was lower within the ulcer region compared with the surrounding tissue in superficial and mid-depth layers, with this difference decreasing at greater depths. OCT angiography further demonstrated spatial parallelism between lymphatic vessel networks and blood vessels in both healthy and ulcerated tissues. These findings demonstrate that OAC-enhanced OCT enables non-invasive, depth-resolved visualization and quantification of oral lymphatic vessel networks, providing reference characteristics for normal oral mucosa and revealing region and pathology-associated lymphatic alterations relevant to inflammatory conditions and early pathological changes.

## 1. Introduction

The oral cavity is the entry point to both the digestive and respiratory systems playing a critical role in overall physiology is essential involvement in speech, mastication, swallowing, and immune defense to maintain healthy daily function [1,2]. Oral squamous cell carcinoma (OSCC) is the most common malignancy of the oral cavity, with global incidence exceeding 389.000 new cases [3]. It carries a high mortality rate when diagnosed at advanced stages [4]. OSCC can arise from previously healthy mucosa or from potentially malignant disorders, and the standard model of oral malignant progression describes a transition from normal epithelium to a thickened white lesion, followed by erythematous change and ultimately a clinically non-healing surface ulceration [5]. Thus, ulcerated morphology is not only a common clinical manifestation but also an integral feature in the early progression of OSCC [6,7]. Early detection can greatly improve patient outcomes, underscoring the need for reliable diagnostic tools capable of identifying pathological changes at an early stage.

The lymphatic system is a key component of immune surveillance, interstitial fluid regulation, and inflammatory response within the oral mucosa. In the mucosal lesions, such as oral ulceration, frequently accompanied by inflammatory lymphocytic infiltration and disruption of the basement membrane zone (BMZ) layer [8]. Localised or band-like lymphocytic aggregates appear at the lesion margin [9]. Such inflammatory lymphatic engagement highlights the dynamic remodelling of the local immune microenvironment. These observations collectively suggest that alterations in lymphatic vessel networks and lymphocyte distribution whether driven by chronic inflammation, epithelial injury with acute inflammation, or early neoplastic transformation may provide diagnostically meaningful signals before malignancy becomes clinically apparent [10–12].

However, the lymphatic structure of oral tissues remains insufficiently visualized and quantified due to technical limitations. Histological studies have shown that the oral mucosa contains abundant T-lymphocytes and Langerhans cells throughout the epithelium and papillary layers, forming a diffuse lymphoid [13]. Lymphatic vessel networks exhibit substantial regional differences across the gingiva, buccal mucosa, and lip even within the dental pulp [14], yet conventional imaging of lymphatic vessel networks typically requires invasive dye injections or biopsies and often suffers from limited spatial resolution [14–16]. The importance in fully understanding lymphatics in detail is pertinent as OSCC frequently spreads through lymphatic and vascular pathways, particularly in regions such as the lower lip, where lymphatic drainage to the submandibular nodes is prominent [17]. Therefore, non-invasive visualization and quantitative assessment of lymphatic vessel networks may provide valuable insight for early detection and disease monitoring.

Optical coherence tomography (OCT) provides non-invasive, high-resolution, real-time imaging of tissue microstructure [18–20], and has become a powerful tool for structural assessment of oral mucosa. One of its functional application, OCT-based angiography (OCTA) can map microvascular networks [21] and has proven valuable for early cancer detection and disease monitoring [22,23]. However, lymphatic vessel networks segmentation is a significant challenge for visualization using OCT-based imaging modalities [15,24], which is because lymph fluid is optically transparent and therefore exhibits extremely low backscattering, making lymphatic structures appear as dark, low-contrast regions within OCT structural images. Depth-related optical attenuation further reduces the visibility of deeper lymphatic vessel networks, impairing segmentation and increasing the likelihood of misclassification. OCT-based lymphangiography has recently demonstrated feasibility for label-free visualization of superficial lymphatics [15,25], and previous studies have further extended this approach to functional lymphatic assessment, including lymph flow measurement [26,27], lymphatic contraction, valve dynamics [28], and longitudinal imaging in disease models [10,29,30]. However, despite these advances, the existing low backscattering of lymph fluid and the non-uniform intensity distribution remains highly challenging in the oral cavity.

To overcome these limitations, optical attenuation coefficient estimation (OAC) has been used as a method to counteract the exponential decay of OCT signal intensity with depth [31–34]. OAC enhances structural contrast by restoring the relative brightness of deeper tissue layers, thereby increasing the distinguishability between low-scattering lymphatic vessel networks and surrounding tissue. This contrast enhancement is crucial for threshold-based segmentation algorithms, enabling more accurate extraction of lymphatic structures across varying depths.

The aim of this study is to apply an OAC-enhanced, threshold-based processing method to OCT structural images of the human oral mucosa, to visualize lymphatic vessel networks and quantitatively measure their depth-resolved density. By improving the contrast of lymphatic features and enabling reliable segmentation, this approach has the potential to support earlier detection of pathological lymphatic alterations and contribute to improved diagnostic assessment in a wide variety of oral conditions including OSCC.

## 2. Material and method

### 2.1. Ethics and participant recruitment

In this study, volumetric OCT microstructure and OCTA datasets were collected by using a 5× objective lens (LSM03, Thorlabs Inc.) and a lab-developed intraoral probe on our lab-built OCT system (Figure), which was described in our previous work [35]. The scan positions are the oral mucosa of the lip, buccal mucosa and hard palate at up floor from participants. 49 volumes of OCT data were collected from 16 participants, all of the data were collected from the lip, during data acquisition using the 5x lens, 3 participants were found to have ulcerative lesions on the lip. Therefore, additional targeted scans of these ulcer sites were collected for further analysis. 78 volumes of OCT data were collected from 21 participants using the intraoral probe, including 16 hard palate data, 24 lip data, and 38 buccal mucosa data. Each acquisition took ∼5 seconds. This study was reviewed and approved by the Research Ethics Committee of the University of Dundee (UOD-SSREC-RPG-BioEng-2022-003), and the Physical Sciences Ethics Committee of the University of York. All participants provided informed consent prior to entering the laboratory for data acquisition, which also conformed to the tenets of the Declaration of Helsinki.

### 2.2. OCT system

**Fig. 1.**
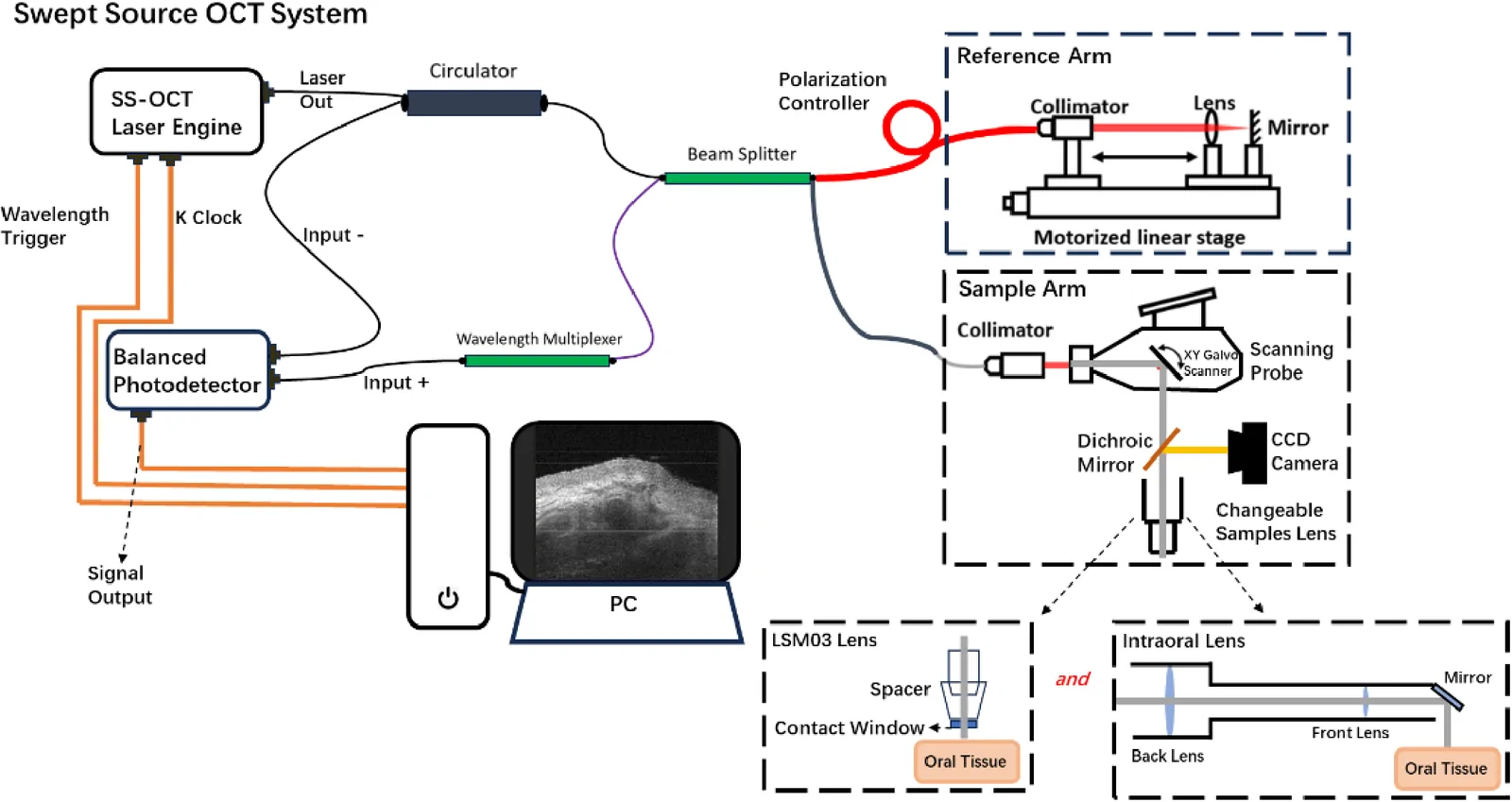
Schematic diagram of the Swept-Source Optical Coherence Tomography (SSOCT) system for oral data acquisitions with changeable samples lens. Lens 1: 5× magnification LSM03 lens from Thorlabs Inc. with a 35 mm focal length. Lens 2: intraoral handheld probe which is a lab-built intraoral lens for difficult-to-reach site data acquisition.

A lab-built, portable Swept-Source OCT (SSOCT) system with different lenses was used in this study, as the imaging system, shown as Fig. 1. This system is a non-invasive, high-resolution, high-speed interferometric imaging device which has been well described in previous papers [36–38]. The system uses a 200 kHz line scanning (A-scan) rate, 1310 nm central wavelength, and 100 nm bandwidth swept laser, providing ∼7.5 µm theoretical axial resolution in air, and the axial conversion coefficient of this SSOCT system is ∼8.74 µm/pixel in air in cross-sectional (B-scan) images. In the system, a beam splitter split the laser beam into reference and sample arms. The system lateral resolution differs from the lenses, for LSM03 is ∼ 19.68 µm in air, and for intraoral lens is ∼ 39.38 µm. For LSM03 lens, each data acquisition involves 600 B-scans, each B-scan consists of 600 A-scans, the field of view was 5.16 × 5.16 mm^2^; and for the intraoral scanning probe, each data acquisition involves 400 B-scans, each B-scan consists of 400 A-scans with 5.2 × 5.2 mm^2^ field of view.

### 2.3. Optimized optical attenuation coefficient estimation

#### 2.3.1. Correction of confocal effect on OCT signal intensity

OCT signal intensity is significantly affected by the focus location, particularly due to the high numerical aperture (NA) of the OCT optics [39]. Therefore, the confocal properties of the OCT system must be taken into account. The confocal bias could be removed by dividing the OCT signal intensity by the axial point spread function (PSF) [40]. The general expression of the confocal axial PSF ( h(z) ) is as follows:

(1) $$\begin{array}{c} h(z)={\left[{\left(\frac{z-z_{cf}}{z_{R}}\right)}^{2}+1\right]}^{-1} \end{array}$$

where z is the signal depth. $z_{cf}$ is the position of the confocal gate, and $z_{R}$ is the “apparent” Rayleigh length is expressed as:

(2) $$\begin{array}{c} z_{R}=\frac{\text{α}\pi nw_{0}^{2}}{\text{λ}} \end{array}$$

where $w_{0}$ is the minimum beam radius, λ is the centre wavelength of the light source, n is the refractive index (assuming average refractive index of tissue ∼1.395 [41] and α is used to distinguish specular reflection ( α=1 ) from diffuse reflection ( α=2 ). In this paper, the $z_{R}\approx 140\;\text{μ}m$ .

#### 2.3.2. Optimized optical attenuation coefficient (OAC) estimation

Depth-resolved estimation methods [42] are commonly employed to compensate for the depth-related signal attenuation inherent in OCT imaging by converting each pixel intensity into a corresponding optical attenuation coefficient (OAC). However, these methods tend to exhibit increasing estimation errors near the maximum imaging depth and do not explicitly account for the OCT noise floor, which can compromise the accuracy of OAC measurements. To address these limitations, an optimal depth-related attenuation coefficient estimation approach [43] was adopted in this study. The OAC (µ(z)) is estimated as:

(3) $$\begin{array}{c} \mu (z)\approx \frac{I(z)}{2\Delta \sum_{i=z+1}^{N}I(i)+\frac{I[N]}{\mu [N]}} \end{array}$$

where ***I(z)*** is the OCT signal intensity at pixel **z**, **Δ** is the axial pixel size (approximately 7.4 µm in this study), and ***N*** is the last pixel of each A-line, assuming that most of the backscattered light has been attenuated by that depth. To estimate ***µ(N)***, 120 pixels of the ranging depth were used as the fitting length, and then fitted with an exponential curve y=αexp⁡(−2μz)+b . The fitted result µ is the average attenuation coefficient of this piece of data and can be considered as the optimal approximation of μ[N] .

**Fig. 2.**
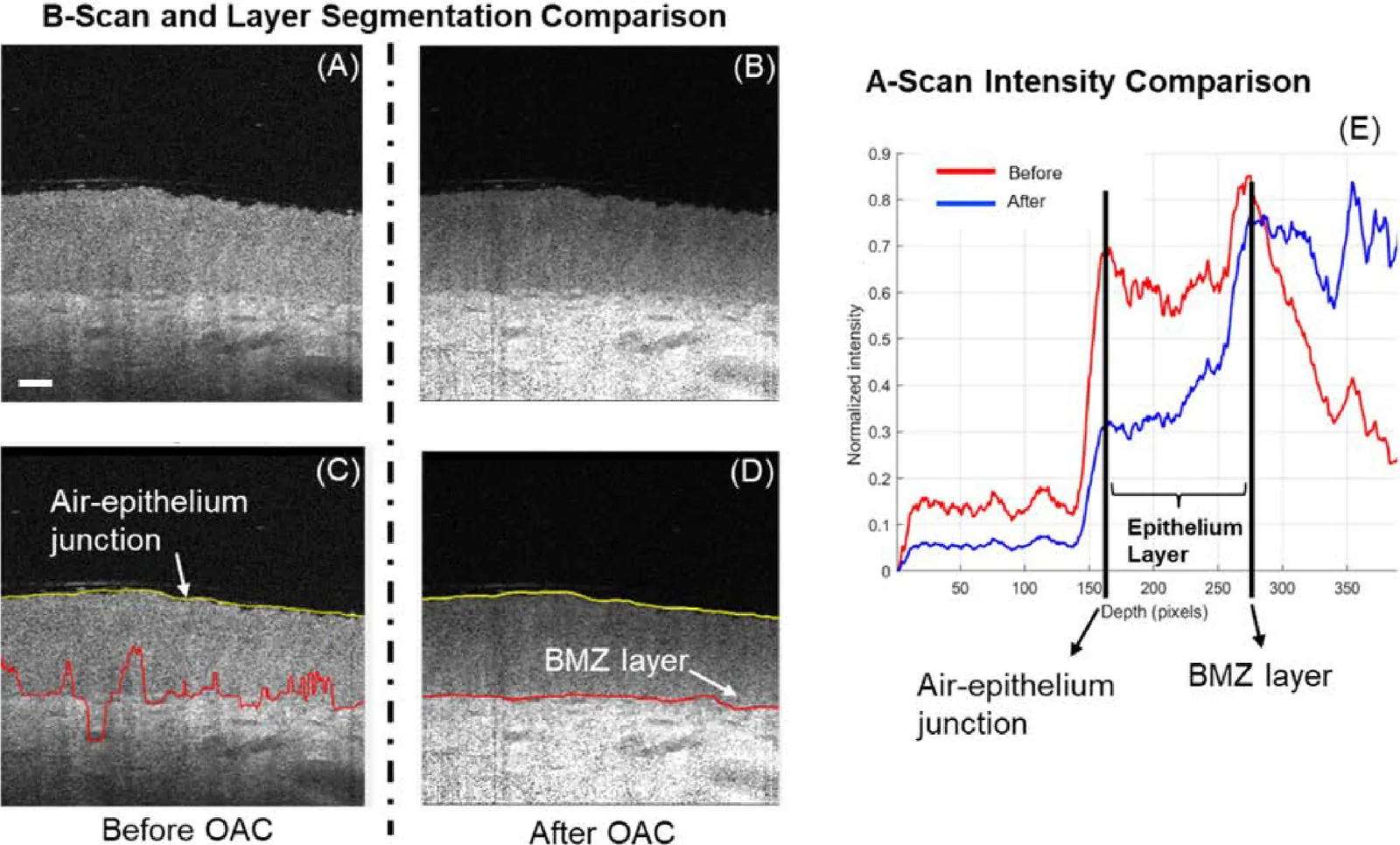
(A) and (B) show a B-Scan result collected from the lip before and after OAC, respectively. (C) and (D) compare BMZ detection before and after OAC based on the most pronounced intensity transitions. Yellow curve: the air-epithelium junction; red curve: the basement membrane zone. (E) illustrates the intensity distribution of one A-Scan line result before and after OAC. Red curve: the original, uncorrected intensity decay with depth; blue curve: the OAC-enhanced signal. White scale bar is 500 µm.

As shown in the A-line intensity distribution in Fig. 2(E), the signal intensity at deeper depths is greatly enhanced after OAC, making the contrast between low-scattering lymphatic vessel networks and the surrounding tissue more distinguishable for threshold-based segmentation. Figure 2 presents a comparison of OCT structural images and their corresponding intensity distributions before and after OAC. After attenuation compensation, the B-frame images exhibit improved contrast in deeper regions, particularly around the basement membrane zone (BMZ), where the intensity drop is most prominent along the A-line. As a result, BMZ localization based on intensity changes becomes significantly more accurate even without manual correction.

### 2.4. Lymphatic network segmentation

Binary segmentation of lymphatic vessel networks was performed using an intensity-based local thresholding strategy for OCT structural images [44]. For each B-scan image, the OCT volume was first intensity-normalized and pre-processed with mild spatial filtering to suppress speckle noise and enhance local contrast. A 16 × 16 pixel-blocks region of interest was then defined along the depth direction, and within this region the image was divided into lateral blocks in which a local threshold was computed. Because lymphatic vessel networks appear as low-scattering, low-intensity regions, pixels below the local threshold were classified as lymphatic vessel networks.

The resulting binary masks from all blocks were merged to form a 3D segmentation. Post-processing with median filtering was applied to remove isolated noise, fill discontinuities, and ensure spatial consistency of the segmented lymphatic network.

For quantitative analysis, the *en-face* projection of the binary volume was partitioned into predefined depth intervals, and lymphatic density was calculated within each depth range as the ratio of segmented low-intensity voxels to the total available tissue voxels. The procedure produced depth-resolved lymphatic density distribution for each scan, enabling quantitative comparison across anatomical sites or pathological conditions.

## 3. Results

### 3.1. Quantification of lymphatic distribution

**Fig. 3.**
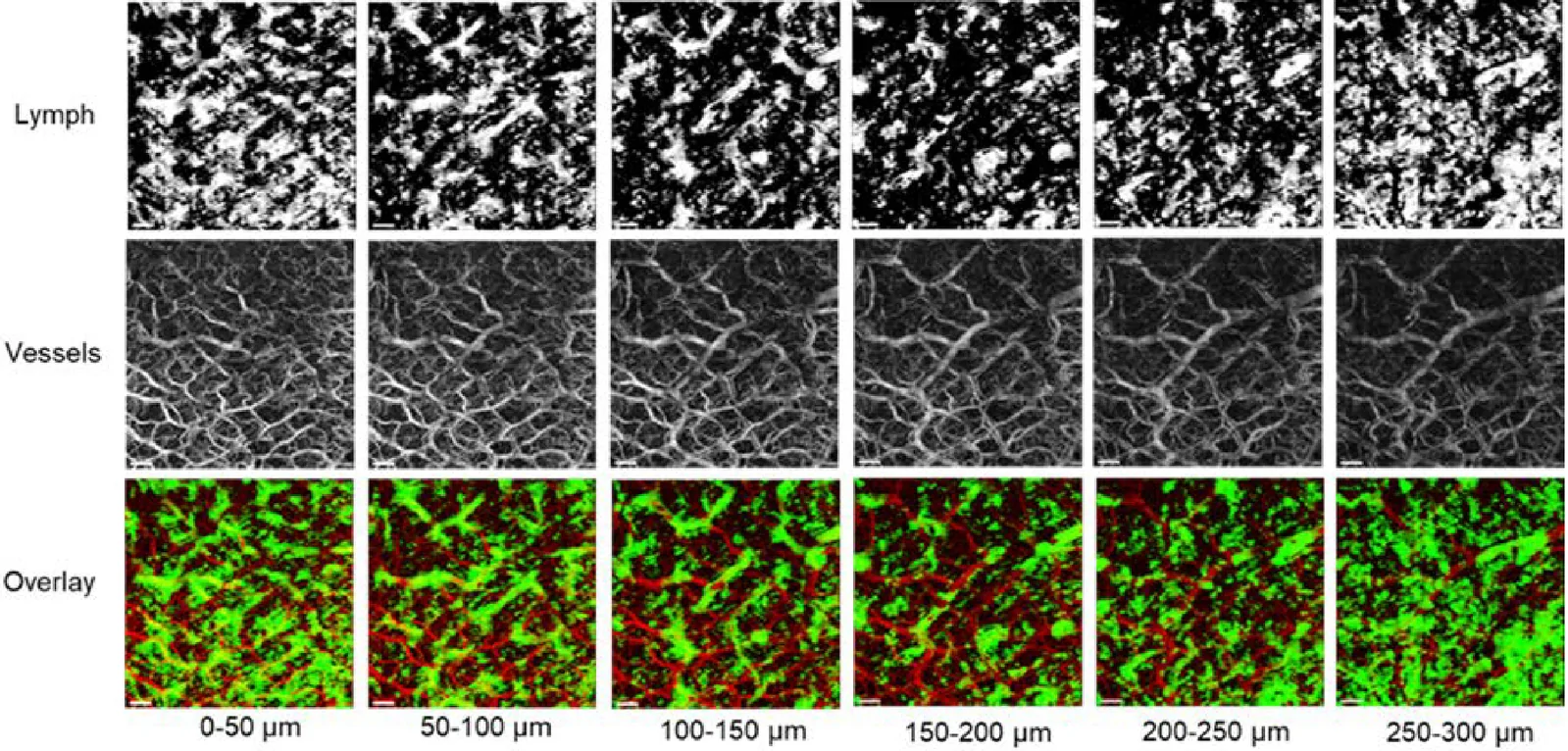
Mean-intensity projection of lymph OCT (top), vessels OCTA (middle) and overlay (bottom) images at depth ranges from 0 to 300 µm, with 50 µm intervals. In the overlay images, the red part is the vessels, green part is the extracted lymphatic network. White scale bar is 500µm.

After applying OAC and flattening each B-scan under the BMZ layer, the lamina propria was isolated for quantitative analysis, as lymphatic vessel networks are absent in the epithelium [17]. Depth-resolved *en-face* projections were generated at 50 µm intervals, and lymphatic density was computed for each depth range. OCTA projections using identical depth steps enabled direct comparison between vascular and lymphatic patterns. As illustrated in Fig. 3, the two microvascular networks frequently appeared in close spatial proximity, demonstrating a consistent spatial parallelism that agrees with anatomical observations reported in previous studies [45]. Using this depth-resolved approach, quantitative comparisons across oral regions revealed distinct lymphatic density distribution in the lip, buccal mucosa, and hard palate.

**Fig. 4.**
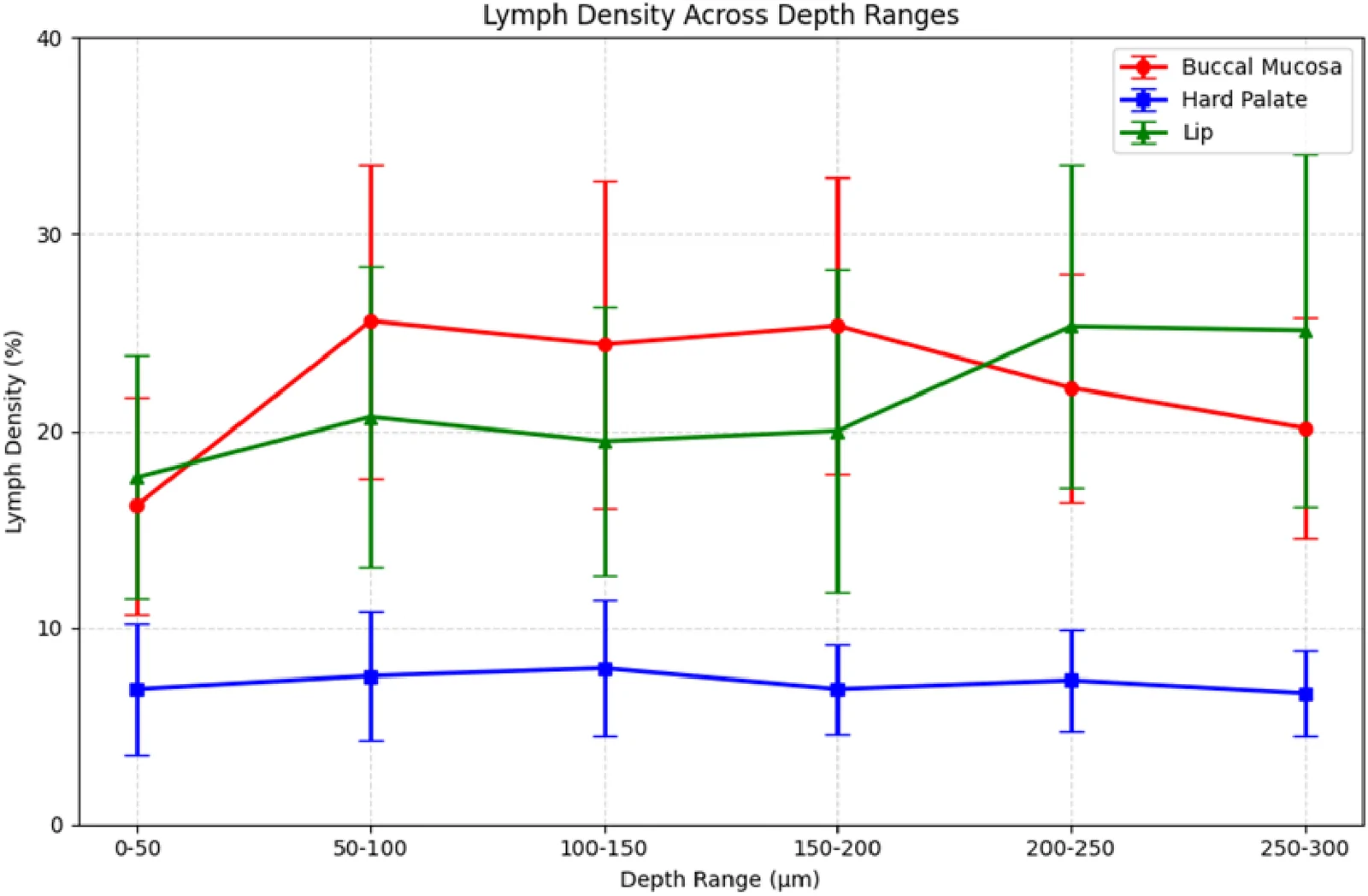
Lymphatic density in different oral regions, red line is the buccal mucosa, green line is the lip, and the blue line is the hard palate.

Quantitative comparisons across oral regions are summarized in Table 1 and visualized in Fig. 4, among all three oral regions, the buccal mucosa exhibited the highest lymphatic density overall, the lip showed intermediate values with higher densities at 200-300 µm, whereas the hard palate consistently showed markedly lower values with little variation.

**Table 1.** Lymphatic density in different oral regions across different depth interval

Depth (µm)	Buccal Mucosa	Hard Palate	Lip
0-50	16.22 ± 5.51%	6.88 ± 3.34%	17.64 ± 6.17%
50-100	25.59 ± 7.97%	7.55 ± 3.32%	20.71 ± 7.67%
100-150	24.40 ± 8.33%	7.94 ± 3.43%	19.47 ± 6.84%
150-200	25.35 ± 7.56%	6.88 ± 2.30%	20.00 ± 8.18%
200-250	22.19 ± 5.77%	7.32 ± 2.61%	25.32 ± 8.19%
250-300	20.15 ± 5.59%	6.67 ± 2.17%	25.12 ± 8.98%

The three oral regions showed different trends in lymphatic density across depth intervals. In the lip, lymphatic density values ranged from ∼18% in the superficial interval (0-50 µm) to ∼26% in the deeper intervals of 200-300 µm. The hard palate consistently showed low lymphatic density across all depth intervals, remaining within a narrow range between 6.67 ± 2.17% and 7.94 ± 3.43%, with limited variation across depths. The buccal mucosa result demonstrated higher lymphatic density values in superficial intervals, with values of 25.59 ± 7.97% at 50-100 µm and 25.35 ± 7.56% at 150-200 µm, followed by lower values at greater depths to about 20.15 ± 5.59% at 250-300 µm.

### 3.2. Lymphatic alterations in ulcerative lesions

**Fig. 5.**
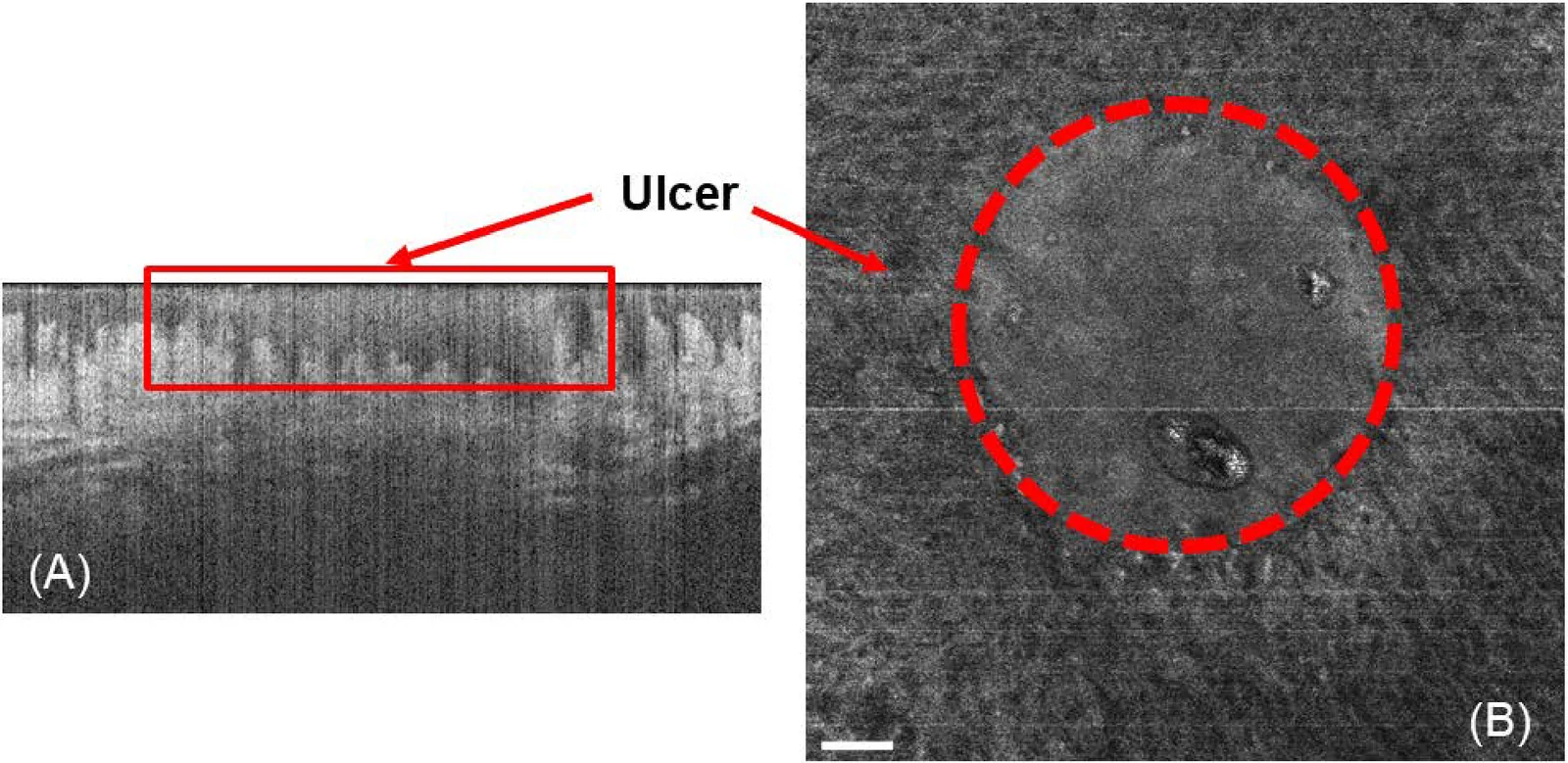
The selected ulcer region is shown, where (A) represents the vascular image obtained from OCT angiography, and (B) shows the en-face structural OCT image. White scale bar is 500 µm.

**Fig. 6.**
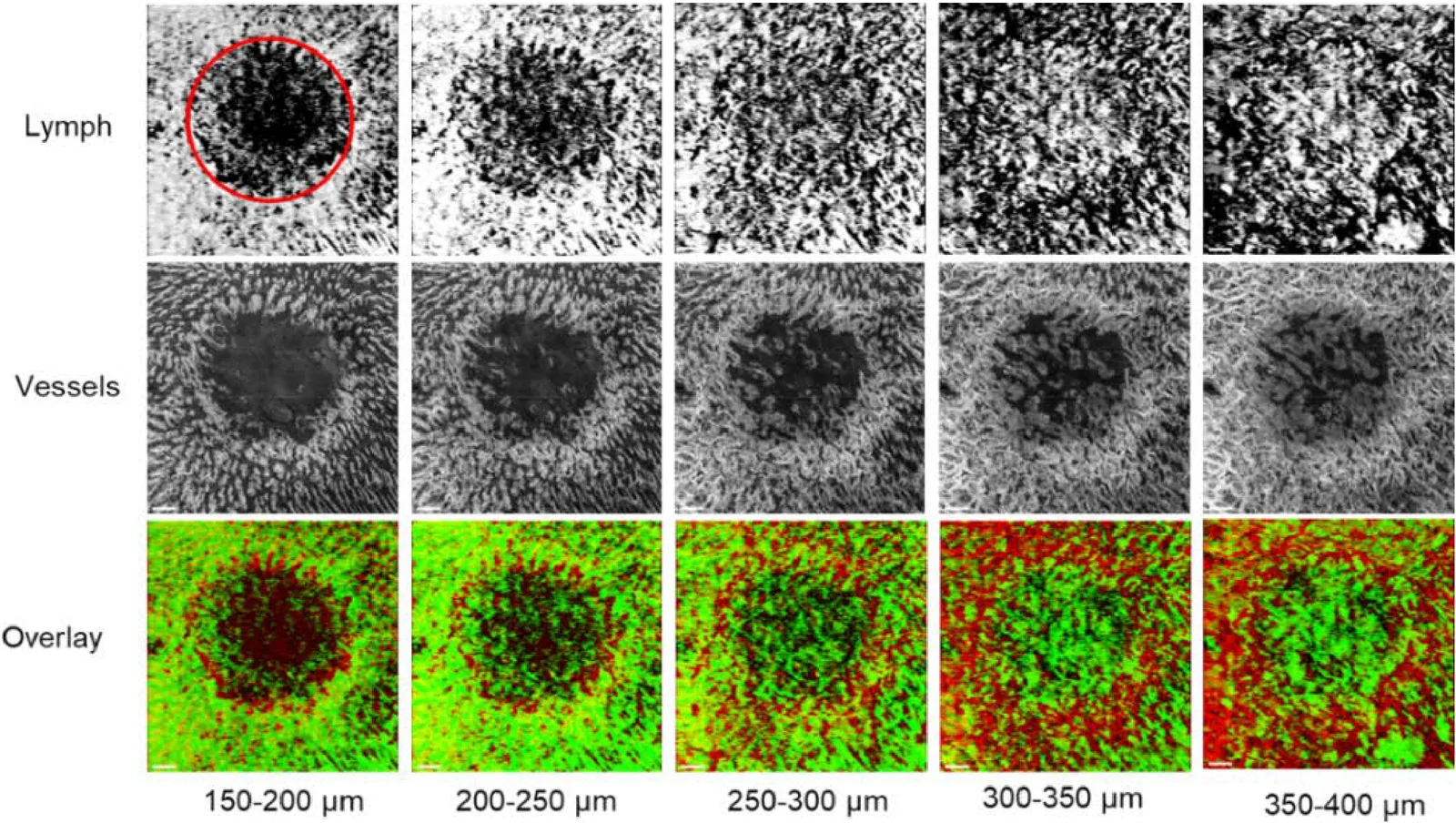
Mean-intensity projection of lymph OCT (top), vessels OCTA (middle) and overlay (bottom) images for one ulcer data. The red circle indicates the area of the ulcer. In the overlay images, the red part is the vessels, green part is the extracted lymphatic network. White scale bar is 500µm.

In participants with ulcerative lesions, the region of interest was selected to include areas containing vasculature, lymphatic vessel networks, and ulcer boundaries, as illustrated in Fig. 5. Depth-resolved projections from a representative case are shown in Fig. 6, demonstrating the combined visualization of structural lymphatics and vasculature.

**Fig. 7.**
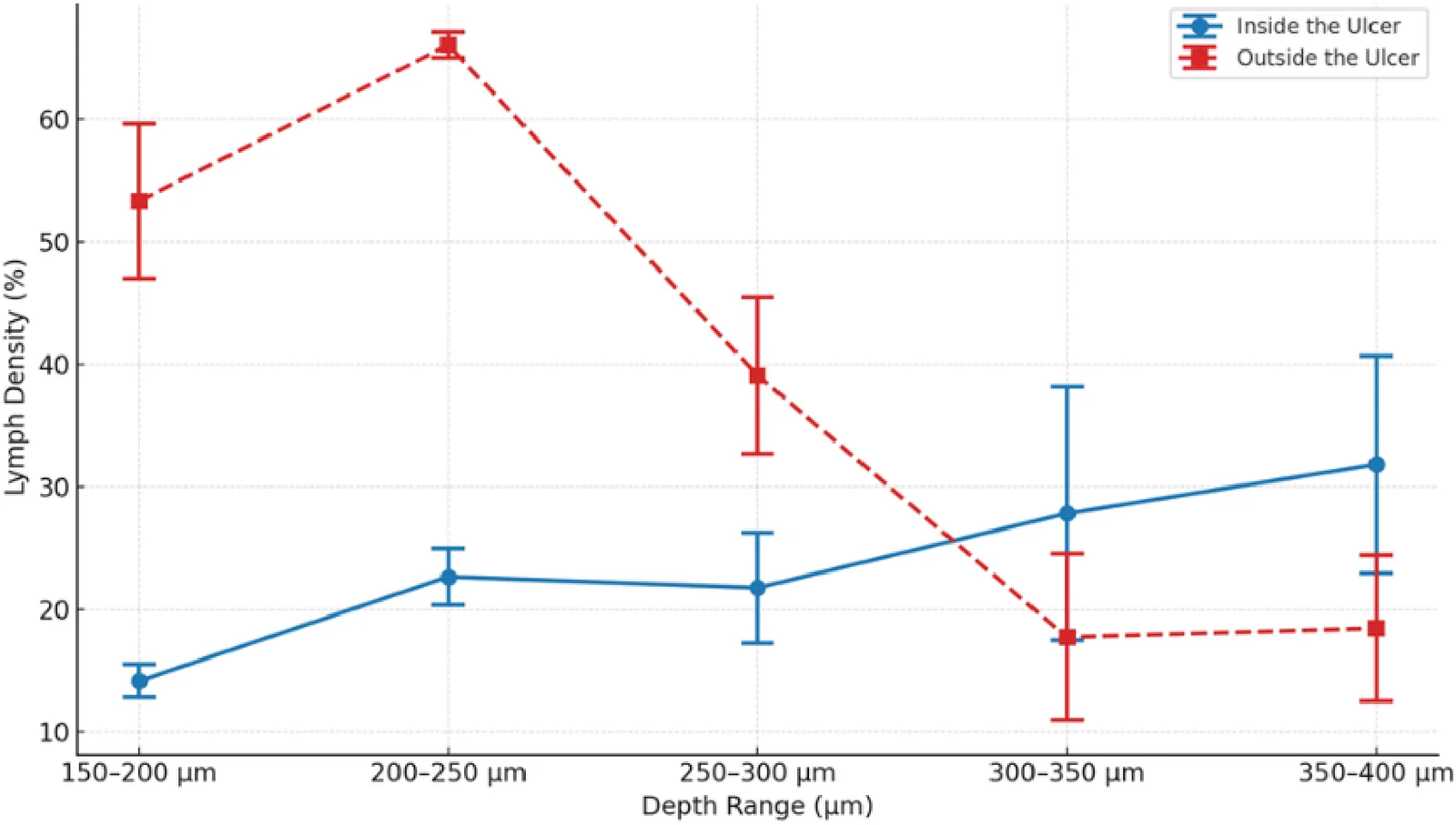
Lymphatic density inside and outside the ulcer region, red dotted line stands for the densities outside the ulcer, and the blue line stands for the densities inside the ulcer.

Lymphatic density was quantified separately inside and outside each ulcer, and the results are summarized in Fig. 7. Across the 150-300 µm depth range, the outside of the ulcer region consistently exhibited higher lymphatic density than the ulcer interior. With increasing depth, this difference diminished, and both curves converged beyond approximately 300 µm. These findings indicate that ulcer-associated alterations predominantly affect the superficial and mid-lamina propria, whereas deeper lymphatic vessel networks remain comparatively preserved.

Across all datasets, this study demonstrates the feasibility of using OAC-enhanced OCT to perform depth-resolved visualization and quantification of lymphatic vessel networks in the oral mucosa. In normal mucosa tissue, the lymphatic density distribution changes across different oral sites. In ulcerative lesions, OCT detected clear reductions in lymphatic density within the ulcer interior relative to surrounding tissue, particularly in the superficial and mid-lamina propria. Additionally, the consistent spatial parallelism observed between lymphatic channels and local vasculature reinforces the biological validity of the segmented structures. Together, these findings highlight the ability of depth-resolved OCT to visualize and quantify normal and pathological lymphatic patterns across the oral mucosa and provide reference characteristics for future structural and disease related assessments.

## 4. Discussion

Oral lymphatic vessels play a critical role in maintaining tissue fluid homeostasis, and they are also essential for immune cell trafficking [46]. Quantitative assessment of the lymphatic network therefore has the potential to facilitate early diagnosis and monitoring of oral pathological conditions. In this study, we demonstrated the use of OCT and OCTA to visualize and quantitatively assess the lymphatic network in in vivo human healthy and abnormal oral cavities. Lymphatic network density was quantified across depth ranges at three intraoral sites, involving buccal mucosa, lip and hard palate. In addition, an analysis of a benign labial ulcer indicated that the density differed from those in the healthy tissues.

Although direct histological validation was not performed in this study, the interpretation of these low-scattering structures as lymphatic vessels is supported by their anatomical location and morphology. Other hypoechoic structures are less consistent with the observed features: normal oral mucosa does not contain hair follicles [47]; collagen bundles are typically highly reflective [48]; nerves show internal fascicular texture rather than an empty luminal space [49]; and minor salivary gland ducts are usually smoother, epithelial-lined, and oriented toward the mucosal surface [50,51]. By contrast, lymphatic vessels are typically irregular or tortuous channels located superficially within the lamina propria or submucosal connective tissue [27], which is consistent with the findings of this study. Nevertheless, histological confirmation remains necessary in future studies.

The descriptive patterns observed in this study may reflect anatomical differences between oral mucosal regions. The hard palate is a highly keratinized and densely fibrous masticatory mucosa that is tightly bound to the underlying bone, which may contribute to the lower mean density of lymphatic vessel-like structures observed in this region [52], In contrast, softer mucosal regions such as the lip and buccal mucosa contain looser and more extensible connective tissue, which may support a more prominent superficial lymphatic network [14,53,54]. However, these interpretations should be treated cautiously, as the present study did not include histological validation or formal statistical comparison between regions.

The observed spatial relationship between lymphatic structures and OCTA-derived vasculature supports a potential anatomical association between these two microvascular systems, as also reported in previous lymphangiography studies [26,27]. Moreover, the depth-wise lymphatic density distribution observed in the oral mucosa are broadly consistent with prior OCT lymphangiography studies reporting lymphatic vessel networks in different tissues. In these studies, superficial lymphatics appear smaller and loose, whereas deeper networks tend to be larger and more interconnected [15].

The primary limitation in OCT-based lymphangiography is the depth-related loss of contrast caused by optical attenuation rather than noise-related Signals-to-Noises Ratio (SNR) degradation [24]. The purpose of the OAC step is to compensate for the exponential attenuation of the OCT signal with depth, thereby restoring the expected brightness of deeper structures and enhancing the contrast between low scattering lymphatic vessel networks and the surrounding tissue, which facilitates threshold-based segmentation. However, OAC cannot compensate for the intrinsic noise-related limitations of SNR because attenuation and noise are from different mechanisms [55]. Depth-related optical attenuation is a deterministic loss of signal energy that can be mathematically corrected [32], whereas SNR loss arises from random noise sources such as speckle, shot noise, and detector noise that are independent of depth. As a result, OAC improves contrast but cannot suppress random noise or recover information lost to noise, and therefore does not address SNR limitations [31,33,34].

Beyond the technical considerations, the biological findings from this study provide additional insights. During the healing process of an ulcer lesion, the local lymphatic system gradually transitions from a damaged or abnormal state to a relatively normal structural and functional condition [56,57]. Therefore, sequential monitoring of the ulcerated mucosa may serve to significantly enhance the accuracy of lymphatic segmentation and identification. Our results also show clear differences in lymphatic distribution inside and outside the ulcer region with depth increasing, the distribution tends to be more consistent. Further extended monitoring of individual ulcers and the surrounding region could improve our understanding of how the lymphatic structure within ulcerated tissue differs not only spatially from that of normal regions but also whether if follows a distinct temporal remodelling trend during healing.

## 5. Conclusion

This study purposed an advanced method for in vivo assessment of lymphatic structures in the oral cavity, including buccal mucosa, lip and hard palate. The lymphatic network was automatically segmented via optimized OAC and intensity-based threshold methods across depth ranges and then their density was accessed. OCT further revealed distinguishable lymphatic alterations between the ulcer interior and surrounding tissue. These findings support the capability of OCT to analyze oral lymphatic vessel networks and provide reference values that may assist future evaluations of normal mucosa and early pathological changes.

## Data Availability

The data that support the findings of this study are available on request from the corresponding author. The data are not publicly available due to privacy or ethical restrictions.
